# Screening of Candidate Effectors from *Magnaporthe oryzae* by In Vitro Secretomic Analysis

**DOI:** 10.3390/ijms24043189

**Published:** 2023-02-06

**Authors:** Guanjun Li, Qingchuan Shi, Yanqiu He, Jie Zhu, Mingluan Zhong, Lingjie Tong, Huaping Li, Yanfang Nie, Yunfeng Li

**Affiliations:** 1 Guangdong Province Key Laboratory of Microbial Signals and Disease Control, College of Plant Protection, South China Agricultural University, Guangzhou 510642, China; 2 Institute of Plant Protection and Agro-Products Safety, Anhui Academy of Agricultural Sciences, Hefei 230031, China; 3 College of Materials and Energy, South China Agricultural University, Guangzhou 510642, China

**Keywords:** *Magnaporthe oryzae*, secretome, shotgun, effectors, bioinformatics

## Abstract

*Magnaporthe oryzae* is the causal agent of rice blast, one of the most serious diseases of rice worldwide. Secreted proteins play essential roles during a *M. oryzae*–rice interaction. Although much progress has been made in recent decades, it is still necessary to systematically explore *M. oryzae*-secreted proteins and to analyze their functions. This study employs a shotgun-based proteomic analysis to investigate the in vitro secretome of *M. oryzae* by spraying fungus conidia onto the PVDF membrane to mimic the early stages of infection, during which 3315 non-redundant secreted proteins were identified. Among these proteins, 9.6% (319) and 24.7% (818) are classified as classically or non-classically secreted proteins, while the remaining 1988 proteins (60.0%) are secreted through currently unknown secretory pathway. Functional characteristics analysis show that 257 (7.8%) and 90 (2.7%) secreted proteins are annotated as CAZymes and candidate effectors, respectively. Eighteen candidate effectors are selected for further experimental validation. All 18 genes encoding candidate effectors are significantly up- or down-regulated during the early infection process. Sixteen of the eighteen candidate effectors cause the suppression of BAX-mediated cell death in *Nicotiana benthamiana* by using an *Agrobacterium*-mediated transient expression assay, suggesting their involvement in pathogenicity related to secretion effectors. Our results provide high-quality experimental secretome data of *M. oryzae* and will expand our knowledge on the molecular mechanisms of *M. oryzae* pathogenesis.

## 1. Introduction

The filamentous fungus *Magnaporthe oryzae* causes rice blast, one of the most destructive diseases of cultivated rice in the world [1]. Fungicide application and development of blast-resistant rice varieties are the main approaches for combating the disease [2]. However, fungicides application is not always effective and has usually led to a harmful effect on the environment [3]. The use of resistant rice varieties is a well-known aspect of integrated disease management; however, this control procedure is limited due to rapid evolution of virulence genes in *M. oryzae* and consistent emergence of new pathovars [4].

*M. oryzae* is a hemibiotrophic fungus that establishes a biotrophic interaction with rice at the first 72 h of infection [5,6]. During the host infection process, *M. oryzae* develops appressoria and penetration pegs to penetrate the leaf cuticle in the biotrophic infection phase [7]. *M. oryzae* secretes an arsenal of proteins to alter host cellular defense processes during leaf invasion that facilitate fungal infection and disease development. Recent studies have shown that some secreted proteins are required for fungal virulence and play important roles in the *M. oryzae*–rice interaction during different infection stages [8]. For example, SLP1, a secreted LysM protein, can act as a suppressor of chitin-triggered defense response and determines the progression of rice blast disease [9]. MoHrip1 secreted during the vegetative growth stage was responsible for the penetration, invasive expansion, and further full virulence of *M. oryzae* [10]. MoAa91, secreted from appressoria, can facilitate the chitin-binding process and suppress host immunity [11]. An avirulence effector AvrPiz-t targets the rice E3 ligases APIP6 and APIP10, interacts with the bZIP-type transcription factor APIP5, suppresses PAMP-triggered immunity in rice, and enhances susceptibility to *M. oryzae* [12,13]. BAS1-4, MSP1, and Rbf1 have been successively identified as virulence factors to promote fungus growth and to facilitate fungal invasion [14,15,16].

In recent years, the secretome of *M. oryzae* has been widely investigated using high-throughput computational and *experimental* techniques. Genome-wide predictions of *M. oryzae* secretome have been performed with bioinformatics tools, revealing from 739 to 1732 putative secreted proteins [17,18,19]. These computational methods to predict fungal secretomes based on sequence and genomic information is a challenging process because of the missing sequencing information [20]. Therefore, an analysis of experimentally determined secretomes instead of computationally predicted secretomes will be highly valuable for discovering novel secreted proteins that lack the canonical secretory pathway unrecognized by current prediction software [21,22]. Wang et al. [23] used a comparative 2DE-based proteomics approach to investigate *M. oryzae* secretome in response to nitrogen starvation and identified 85 differentially expressed proteins by MALDI-TOF-MS and μLC-ESI-MS/MS. Jung et al. [24] established an in vitro system to perform *M. oryzae* secretome by mimicking the early stages of infection and identified the first secretome (53 non-redundant proteins) of *M. oryzae*. Kim et al. [25] investigated the proteomic profiles of rice leaves infected with *M. oryzae* and identified a total of 441 secreted proteins derived from *M. oryzae*. The above experimental studies have increased our knowledge on *M. oryzae* secretome and the early interactions between *M. oryzae* and rice. However, these early studies mainly relied upon 2DE-based proteomic technologies, which can only identify a small number of secreted proteins. 

To have a better view of the *M. oryzae* secretome, the secreted protein profiles in *M. oryzae* were conducted during conidial germination by germinated conidia in PVDF membrane to mimic the early stages of the *M. oryzae*–rice interaction. A shotgun-based proteomics approach was employed to identify the secreted proteins of *M. oryzae*, followed by construction of the high-quality secretome database of *M. oryzae*. Candidate effectors predicted by bioinformatics analysis were verified by using the *Agrobacterium*-mediated transient expression system and qRT-PCR. Our data will serve as baseline information during the future discovery of proteins secreted by *M. oryzae*. Furthermore, this study will facilitate an understanding of the molecular basis of *M. oryzae*–rice interactions.

## 2. Results

### 2.1. Preparation of M. oryzae Secretome

The aim of this work was to analyze *M. oryzae* secretome using a shotgun-based approach in order to better understand the molecular mechanism of the early stages of *M. oryzae* infection. To mimic the *M. oryzae*–rice interaction and to maximize the number of secreted proteins, conidia were allowed to germinate on PVDF membrane, which provided hydrophobic conditions for *M. oryzae*. Previous studies showed that PVDF membrane can be used as suitable platforms for developing the secretome of *M. oryzae* during the early infection process [24]. In this study, two distinct stages of *M. oryzae* conidial germination were selected for secretome analysis, corresponding to germ-tube growth (8 h) and appressoria formation (24 h) (Figure S1). The secreted proteins were collected mainly by centrifugation and ultrafiltration. A total of 180 ± 24 μg secreted proteins of each treatment were obtained from 10 mL of elution buffer. The efficiency and reproducibility were tested by subjecting the total secreted proteins to SDS-PAGE. The representative gel showed similar protein bands (Figure S2).

### 2.2. Shotgun Analysis of M. oryzae Secretomes

To investigate *M. oryzae* secretome, we combined the secreted proteins collected from 8 h and 24 h as one sample. Three independent biological samples were analyzed using LC-MS/MS. In total, 3315 non-redundant secreted proteins were identified (Table S1). The majority of detected proteins were similar among all the replicates, indicating high reproducibility and reliability of our analysis (Figure S3). The length of the secreted proteins ranged from 24 to 5707 amino acids in *M. oryzae*, mainly distributed in 100–600 amino acids (Figure S4).

### 2.3. In Silico Analysis of M. oryzae Secretome

**Figure 1:**
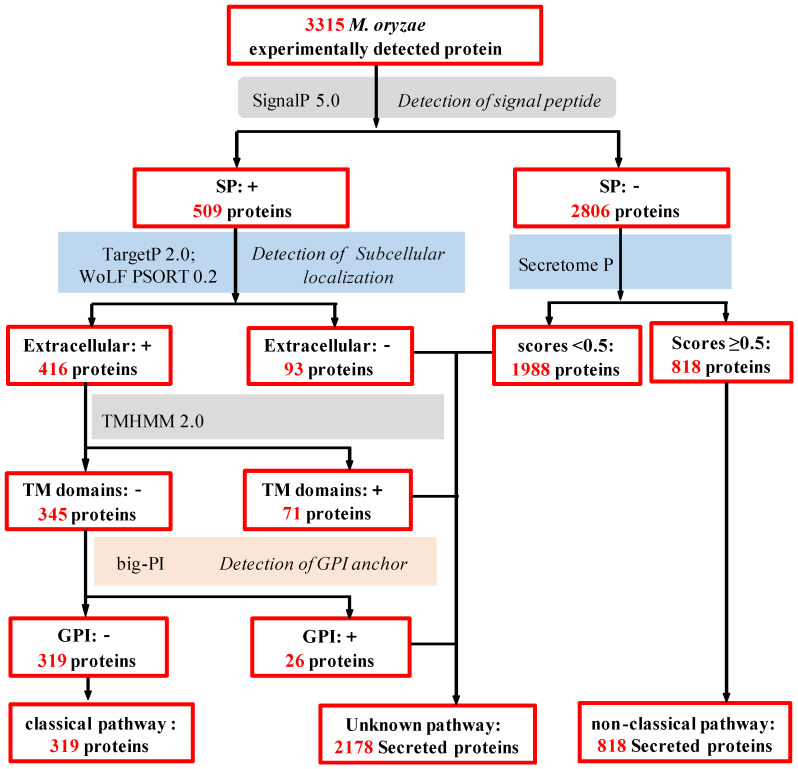
The bioinformatics pipelines used to predict the *M. oryzae* secretomes.

To analyze the secretory pathways of these secreted proteins, we performed silicon wafer analysis of the *M. oryzae* secretory group using state-of-the-art protein prediction tools (Figure 1). Firstly, 3315 *M. oryzae*-secreted proteins were observed for the presence of signal peptide (SP; by SignalP 6.0). The results showed that 509 proteins (15.4%) contained N-terminal signal peptides, and 2806 proteins (84.6%) did not contain N-terminal signal peptide. The subcellular localizations were predicted by amino acid sequence searching of the 509 amino acid sequences using the WoLF PSORT and TargetP 2.0 Server. The prediction analysis revealed that 416 proteins were secreted, whereas 93 proteins were not secreted and were located in plasma membrane, cytoplasm, peroxisome, nucleus, and Golgi apparatus (Figure S5A). The transmembrane domain analysis of 416 proteins using TMHMM Server V2.0 software suggested that 345 proteins contained no transmembrane domains, 65 proteins contained transmembrane domain, 4 proteins contained 2 transmembrane domains, and 2 proteins contained more than 2 transmembrane domains (Figure S5B). Big-Pi Predictor was used to analyze the GPI anchor sites of the above proteins without transmembrane helices. The results showed the absence of GPI anchor sites in 319 proteins and their presence in 26 proteins. Based on the above analysis, 319 of 3315 *M. oryzae* secreted proteins were consistent with the characteristics of classical secreted proteins (Figure 1 and Table S2).

Different from the classical secretion pathway, the unconventional secretion pathways of regulatory proteins are diverse and depend on the type of secreted protein, the stimulation of the cell, and the cell type [26]. In this study, SecretomeP 3.0 was used to predict the remaining 2806 protein sequences without signal peptides, and the results showed that a total of 818 proteins were predicted to be non-classical secreted pathway proteins. Therefore, the other 1988 secreted proteins in the secreted protein database are neither classical secreted pathway proteins nor non-classical secreted pathway proteins (Figure S5C and Table S2), indicating that *M. oryzae* may possess various secretory pathways, including classical and non-classical secretory pathways, and previously unknown secretory pathways.

### 2.4. Functional Annotation and Classification of the Secreted Proteins

**Figure 2:**
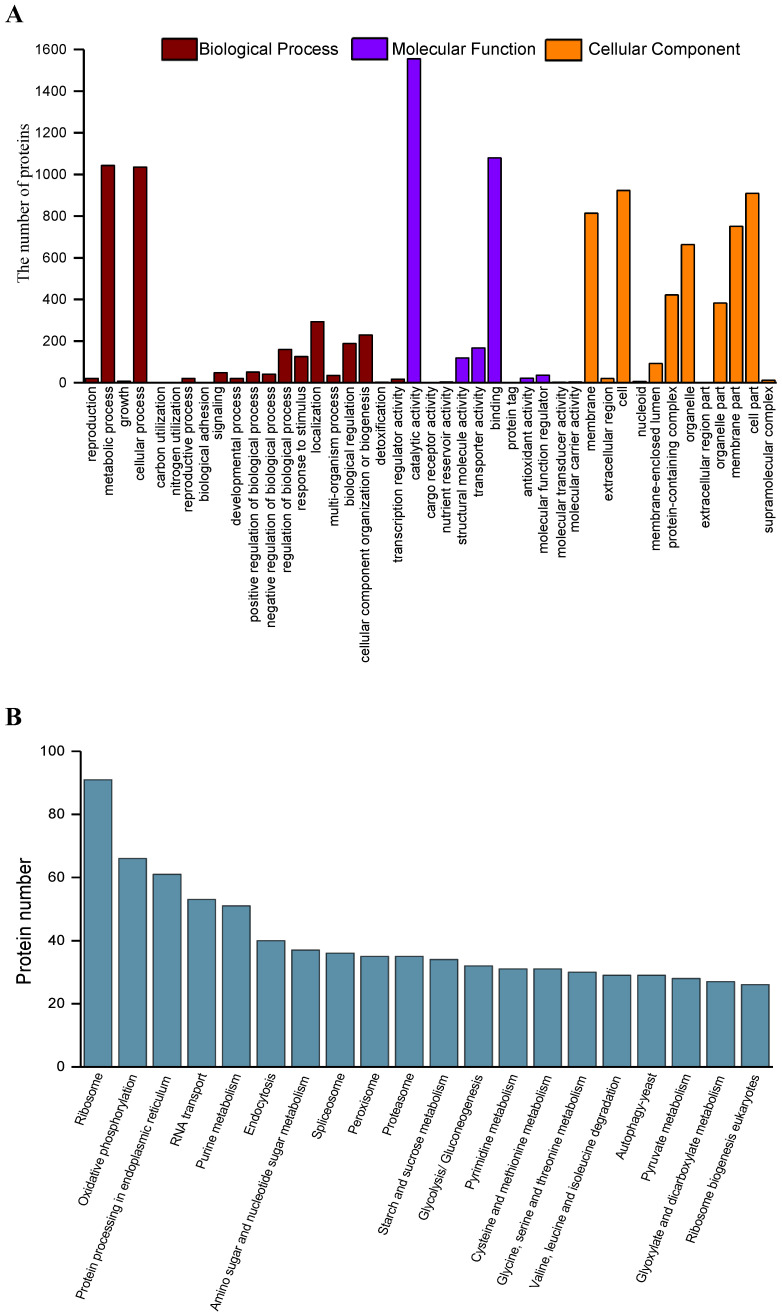

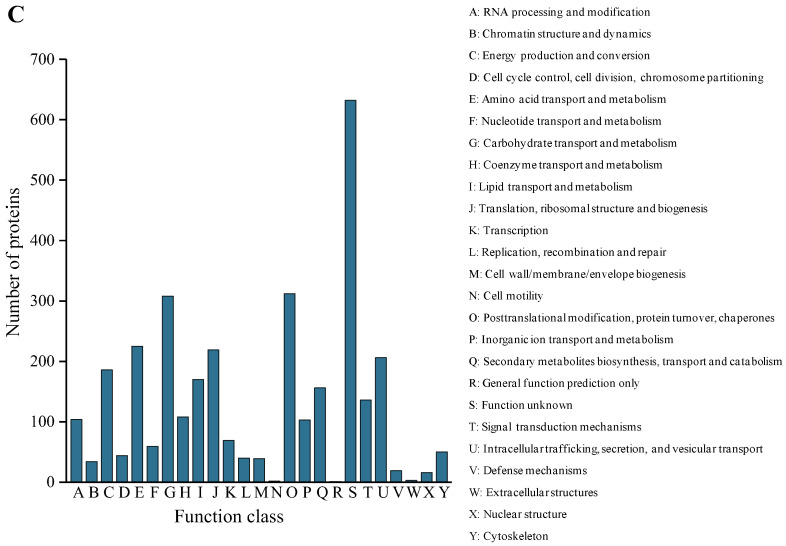
Functional annotations of *M. oryzae* secretome. (**A**) COG functional categories; (**B**) GO molecular function terms; (**C**) KEGG pathways. The top 20 items in each type of functional annotations were shown.

Functional annotation of the 3315 secreted proteins was carried out using EggNOG-mapper. In total, 3033 (91.5%) secreted proteins with significant homology were assigned to the appropriate COG clusters, which were further grouped to 25 functional categories (Figure 2A). The cluster ‘function unknown’ (S, 632) represented the largest group (indicating that a considerable fraction of the secreted protein was functionally uncharacterized), followed by ‘posttranslational modification, protein turnover, chaperones’ (O, 312); ‘carbohydrate transport and metabolism’ (G, 308); ‘amino acid transport and metabolism’ (E, 225); ‘translation, ribosomal structure and biogenesis’ (J, 219); and ‘intracellular trafficking, secretion and vesicular transport’ (U, 206) (Figure 2A). The major prevalence of peptidases in category O pointed towards their involvement in *M. oryzae* pathogenicity. The presence of carbohydrate active enzymes (CAZymes) in category G confirmed their involvement in pathogen–host interactions. The category U contains intracellular transport, and secretion and vesicle transport, which are important for effector secretion leading to its pathogenicity.

Gene Ontology (GO) analysis was carried out to identify the functions of the secreted proteins. Based on sequence homology, the 2668 (80.5%) proteins were categorized into 43 functional groups (Figure 2B). In each of the three main categories (biological process, cellular component, and molecular function) of the GO classification, the major subcategories were as follows: three subcategories for biological process (‘cellular process’, ‘metabolic process’, and ‘localization’); three subcategories for molecular function (‘binding’, ‘transporter activity’, and ‘catalytic activity’); and five subcategories for cellular component function (‘membrane’, ‘cell’, ‘protein-containing complex’, ‘organelle’, and ‘organelle part’). Only a few proteins were clustered in terms of ‘carbon utilization’, ‘nitrogen utilization’, ‘biological adhesion’, ‘detoxification’, ‘cargo receptor activity’, ‘nutrient reservoir activity’, ‘protein tag’, ‘molecular transducer activity‘, ‘molecular carrier activity’, and ‘extracellular region part’ (Figure 2B).

All secreted protein sequences from the *M. oryzae* secretome were subjected to KEGG pathway annotation. In total, 1843 (55.6%) proteins were annotated, which were functionally classified into 111 KEGG pathways. Of all the identified pathways, proteins involved in ribosomes were the most abundant, followed by proteins involved in oxidative phosphorylation, proteins involved in endoplasmic reticulum protein processing, and proteins involved in RNA transport (Figure 2C).

### 2.5. CAZymes and CWDE Analysis of the Secreted Protein

**Figure 3:**
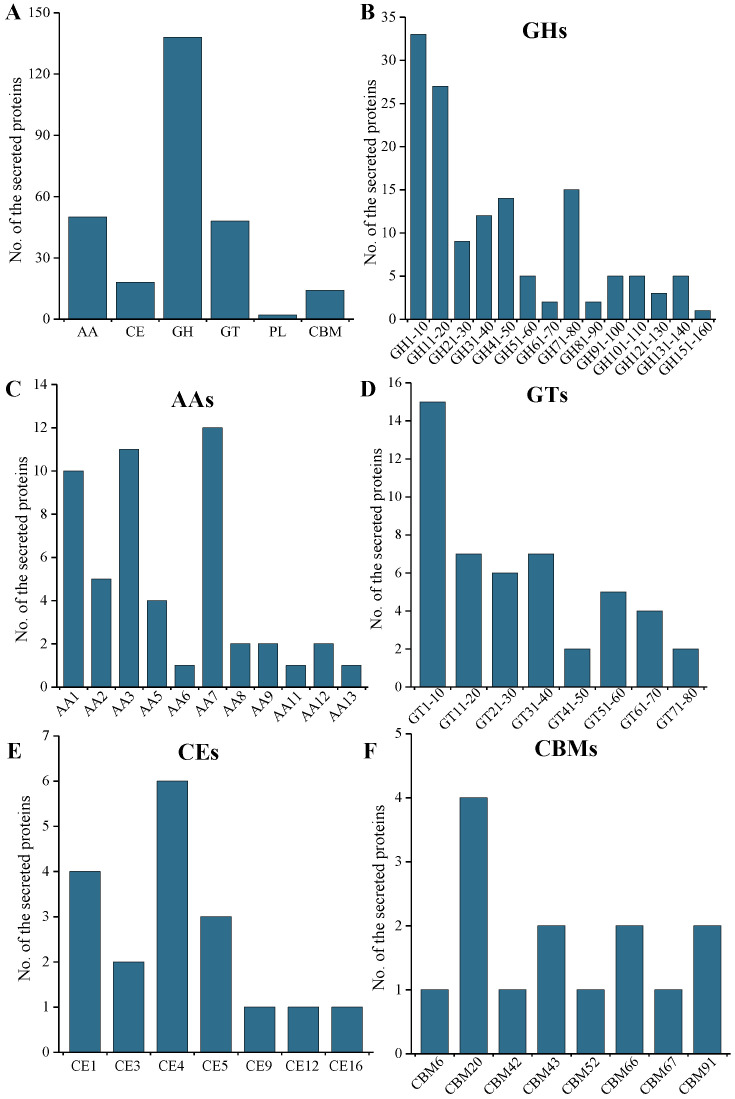
CAZymes proteins and CAZymes families identified in *M. oryzae*. (**A**) Classification of CAZymes; (**B**) composition and contents of the GH family; (**C**) composition and contents of the AA family; (**D**) composition and contents of secreted proteins of the GT family; (**E**) composition and contents of the CE family; (**F**) composition and contents of the CBM family.

The secreted CAZymes by pathogenic fungi can degrade plant cell wall carbohydrates to simple monomers, which can act as carbon sources for fungal invasion [27]. CAZymes commonly include glycoside hydrolases (GHs), glycosyltransferases (GTs), polysaccharide lyases (PLs), carbohydrate-binding modules (CBMs), carbohydrate esterases (CEs), and auxiliary activities (AAs) [28]. A total of 257 CAZymes were identified using the CAZY database, accounting for 7.8% of the total number of *M. oryzae* secretome (Figure 3A). The GH family was the most abundant, with 138 proteins distributed in 46 subfamilies, accounting for 53.7% of the total CAZymes (Figure 3B). The second most abundant class was the AA family and the GT family, containing 50 proteins distributed in 11 AA subfamilies (Figure 3C) and 50 proteins distributed in 23 GT subfamilies, respectively (Figure 3D). In comparison, the number of CE family and CBM family were much smaller (Figure 3E,F), and the PL family contained only two proteins. 

**Figure 4:**
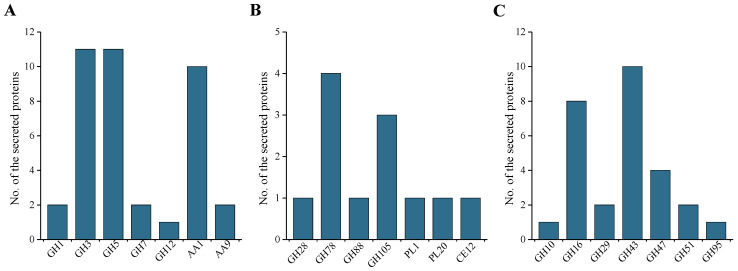
Classification of cell wall degrading enzymes of *M. oryzae*. (**A**), cellulose-degrading enzymes; (**B**), pectin degrading enzymes; (**C**), xylan degrading enzymes.

In many phytopathogenic fungi, some CAZymes act as cell wall-degrading enzymes (CWDEs) to be involved in plant cell wall degradation [29]. In this study, 79 secreted proteins were identified as CWDEs. Among these CWDEs, 39 proteins were identified as cellulose-degrading enzymes, 12 proteins were identified as pectin-degrading enzymes, while 28 proteins were identified as hemicellulose-degrading enzymes (Figure 4). Among these CWDEs, some subfamilies were related to the pathogenicity of fungi. For example, AA9 is involved in the degradation of chitin and cellulose [30], GH3 is involved in the degradation of cellulose and xylan in the plant cell wall, and GH43 participates in the degradation of pectin and xylan [27].

### 2.6. Effector Analysis of M. oryzae Secretome

Previous studies showed that most of the known fungal effectors have the following characteristics: signal peptide, small molecular weight, and rich in cysteine [31]. In this study, we used two different approaches to predict candidate effectors in *M. oryzae* secretome. Of all the classical secreted proteins identified, 59 candidate effectors were obtained with the criteria of amino acid length ≤300 and cysteine number ≥4. EffectorP 3.0 also resulted in 65 candidate effectors. There were 34 common candidate effectors shared by the criteria of the two approaches (Figure S6). Therefore, a total of 90 candidate effectors were obtained by using two approaches, accounting for 28.2% of 319 classical secreted proteins. Of these candidate effectors, 39 were functionally annotated against NCBI database. However, the rest of 51 candidate effectors were annotated as hypothetical proteins. Among these, 47 were predicted to be apoplast effectors, 10 were cytoplasmic effectors, and 8 were classified to be both apoplast and cytoplasmic effectors (Table S3).

### 2.7. qRT-PCR Analysis of Candidate Effectors

**Figure 5:**
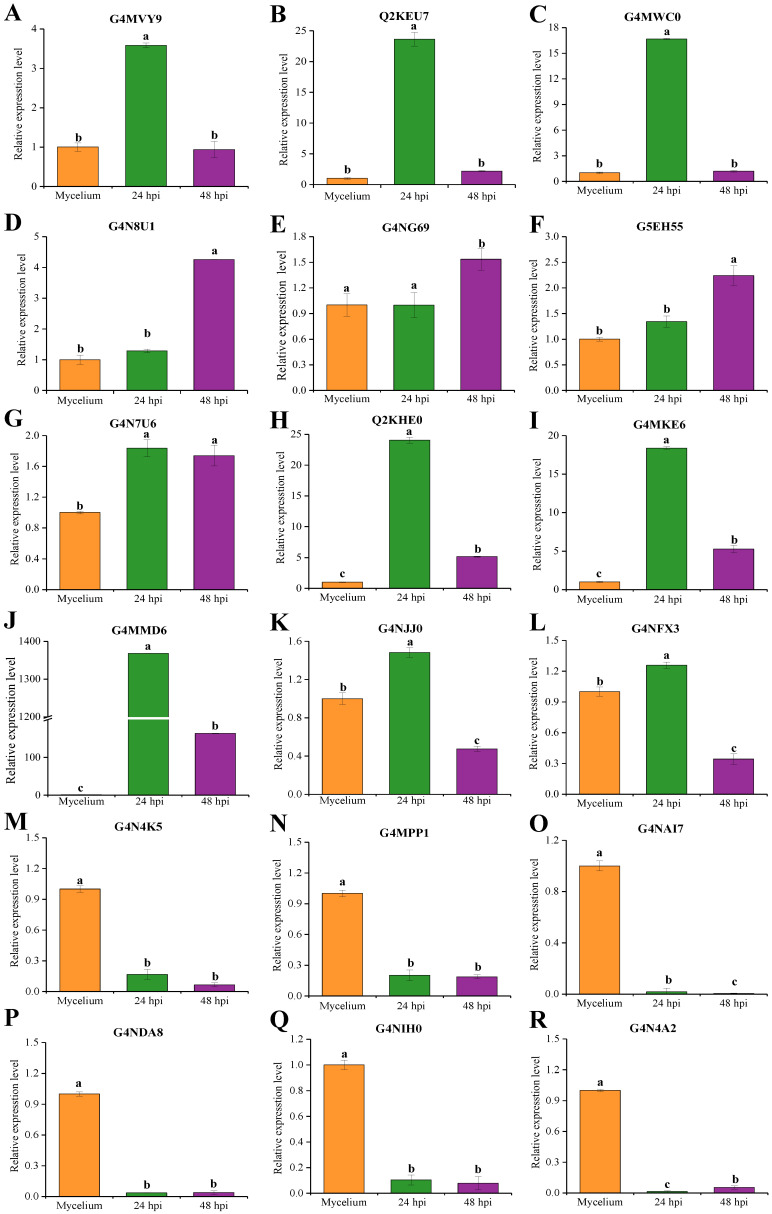
Expression analysis of 18 candidate effector genes in *M. oryzae*-inoculated rice leaves as determined by qRT-PCR. (**A**) G4MVY9; (**B**) Q2KEU7; (**C**) G4MWC0; (**D**) G4N8U1; (**E**) G4NG69; (**F**) G5EH55; (**G**) G4N7U6; (**H**) Q2KHE0; (**I**) G4MKE6; (**J**) G4MMD6; (**K**) G4NJJ0; (**L**) G4NFX3; (**M**) G4N4K5; (**N**) G4MPP1; (**O**) G4NAI7; (**P**) G4NDA8; (**Q**) G4NIH0; and (**R**) G4N4A2. The *M. oryzae* constitutive gene *Moactin* was used as internal reference. Values are the means based on three independent experiments and bars indicate standard deviations. Different letters indicate statistical significance (*p* < 0.05) using Duncan’s new multiple range method.

**Figure 6:**
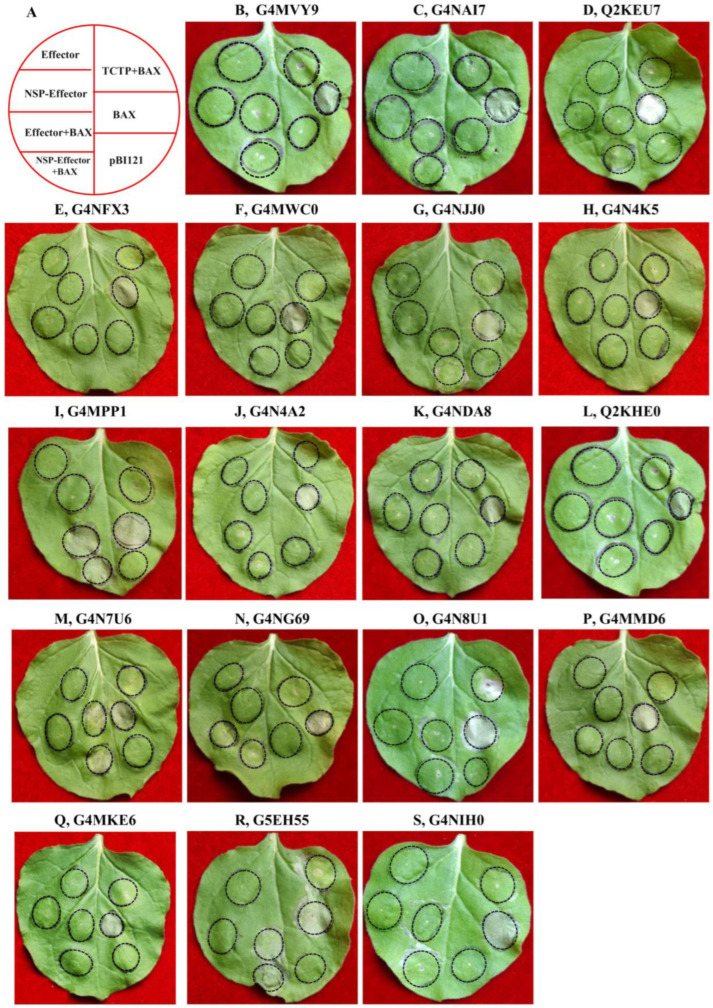
Effects of transient expression of *M. oryzae* candidate effectors on BAX-induced cell death in *N. benthamiana* leaves using agroinfiltration. *N. benthamiana* leaves were infiltrated with *A. tumefaciens* cells carrying pBI121 empty vector, the BAX (Bcl2-associated X protein) gene, the TCTP (translationally controlled tumor protein) gene, or candidate effector genes. BAX was used as positive control, and TCTP was used as negative control. The leaves were photographed at 5 days post-infiltration. (**A**) Schematic diagram showing injection on *N. benthamiana* leaves. (**B**) G4MVY9; (**C**) G4NAI7; (**D**) Q2KEU7; (**E**) G4NFX3; (**F**) G4MWC0; (**G**) G4NJJ0; (**H**) G4N4K5; (**I**) G4MPP1; (**J**) G4N4A2; (**K**) G4NDA8; (**L**) Q2KHE0; (**M**) G4N7U6; (**N**) G4NG69; (**O**) G4N8U1; (**P**) G4MMD6; (**Q**) G4MKE6; (**R**) G5EH55; and (**S**) G4NIH0.

Using *in planta*-based induced expression detection is an important method to evaluate whether candidate effectors are true effectors [32]. Therefore, we randomly selected eighteen genes encoding candidate effectors to determine the expression in *M. oryzae* (the detailed information was listed in Table S4). RNA samples of vegetative mycelia and infected leaves after inoculation with *M. oryzae* conidia at 24 and 48 h were subjected to qRT-PCR analysis to observe the expression levels of abovementioned genes (Figure 5). We compared the expression of genes in rice leaves inoculated with *M. oryzae* or vegetative mycelia and found that (1) the expression of genes encoding G4MVY9, Q2KEU7, and G4MWC0 was significantly up-regulated in infected leaves at 24 h after inoculation (Figure 6A–C); (2) the expression of genes encoding G4N8U1 and G5EH55 was significantly increased at 48 h (Figure 6D,E); (3) the expression of genes encoding G4N7U6, Q2KHE0, G4MMD6, and G4MKE6 was significantly up-regulated at 24 h and 48 h after inoculation (Figure 6G–J); (4) the expression of genes encoding G4NJJ0 and G4NFX3 was significantly increased at 24 h but decreased at 48 h (Figure 6K,L); and (5) the expression of genes encoding G4N4K5, G4MPPP1, G4NAI7, G4NDA8, G4NIH0, and G4N4A2, was significantly down-regulated at 24 h and 48 h (Figure 6M–S). Consistent with our results, Zhang et al. [33] reported that numerous effectors of *M. oryzae* are also highly expressed *in planta*, and their expression is related to histone modification dynamics at H3K27. These results suggest that histone modification dynamics may contribute to the *in planta* gene induction of candidate effectors in *M. oryzae*.

### 2.8. Transient Expression Assays

The ability to suppress BAX-triggered cell death is a well-known technique for the initial screening of fungal candidate effectors [18,34,35]. To evaluate the function of the above candidate effectors, we further selected the above 18 candidate effector genes for transient expression in *N. benthamiana* using an *Agrobacterium*-mediated transformation system. The results showed that 16 of 18 candidate effectors (except for G4MPP1 and G5EH55) significantly suppressed BAX-mediated cell death in *N. benthamiana* leaves after 5 days of infiltration (Figure 5), while no one was found to induce cell death in *N. benthamiana* (Figure S7). Taken together, the results indicate that most candidate effectors can suppress or interfere with plant defense responses, suggesting that the effectors are involved in plant immune suppression.

## 3. Discussion

Phytopathogenic fungi secrete proteins to manipulate the host in order to facilitate colonization and infection, to suppress plant defense, and/or to induce plant cell death [36,37]. Secretomics is a powerful tool to investigate the pathogenicity mechanisms because of its relevance to fungus–plant interactions [38]. Several 2DE-based secreteomic investigations have been performed during the past decade to elucidate rice–*M. oryzae* interactions. In this experimental secretome analysis, a total of 3315 nonredundant proteins were identified by a shotgun-based proteomics approach. It should be noted that the number of secreted proteins was significantly higher than those of other experimental secretome reports, mainly due to the high sensitivity and resolution of shotgun-based approach. Compared with the identified secreted proteins (53 and 441), as reported by Kim et al. [25] and Jung et al. [24], there are shared 39 and 341 proteins in common, respectively, suggesting that our experimental results are reliable. However, functional roles of the identified secreted proteins remain largely unknown and should be further validated by experimental measurements.

During infection, fungal plant pathogens secrete larger numbers of proteins to facilitate colonization [39]. In general, most of the proteins in eukaryote are secreted through the conventional Golgi/ER secretory pathway [21]. However, recent work disclosed unusual type of proteins without signal peptides, known as leaderless secreted proteins (LSPs), which were secreted through the unconventional secretory pathways in fungi [40,41]. More than 50% of the total identified secretome has been identified as LSPs in some fungi [39]. Previous studies also showed that 27.8% of the *M. oryzae* secretome was predicted as LSPs of the *M. oryzae* secretome based on the sequence features of LSPs using SecretomeP [24]. In this study, 818 proteins were identified as LSPs by SecretomeP (scores > 0.5), representing 24.7% of the identified proteins. However, 60.0% (1988 proteins) of the remaining identified proteins, without signal peptides and low Secretome P scores, were not predicted as secreted proteins by these six bioinformatics programs. Consistent with our results, some experimental secretome analysis also showed that many secreted proteins in fungi could not be predicted to be secreted proteins by bioinformatics tools. For example, Wang et al. [23] reported that 17.6% of *M. oryzae* secretome cannot be classified as secreted proteins through a bioinformatics analysis. In *Fusarium oxysporum* f. sp. *cubense* race 1 and race 4, 55.6% and 68.0% of the secretomes were not predicted to be secreted proteins [42,43]. These results suggested that *M. oryzae* potentially possess multiple secretory pathways/mechanisms, including well-characterized Golgi/ER, unconventional secretory mechanisms, or yet unknown secretory mechanisms [20].

In phytopathogenic fungi, CAZymes are involved in the degradation of plant polysaccharide materials to facilitate infection and/or to gain nutrition [44,45]. Some CAZymes are also well-known as CWDEs, mainly including glycoside hydrolases, pectinases, cellulases, and hemicellulase, which act as virulence factors during the infection process [46]. In this study, we identified 257 CAZymes, including 39 cellulose degrading enzymes, 12 pectin degrading enzymes, and 28 hemicellulose degrading enzymes. Previous studies showed that majority of these proteins are tightly regulated and can increase the capacity for efficient nutrient uptake and energy production of *M. oryzae* for proper growth and development [24]. Therefore, we predict that these CAZymes and CWDEs detected in this experimental secretome can be an important reason for establishing a successful infection of *M. oryzae* on rice plants. 

*The hemibiotrophic* fungal pathogen *M. oryzae* secretes a large repertoire of effectors during pathogen and plant interactions. Genome-based bioinformatics analysis predicted that *M. oryzae* secretes about a large number of effectors [18]. Previous studies also identified many well-characterized effectors in *M. oryzae*, including AvrPiz-t [34], Avr-Pita [47], Avr-Pii [19], SLP1 [9], ACE1 [48], Pwl1 [49], Mc69 [50], and BAS1-4 [14], as well as newly characterized effectors such as MoCDIP1-5 [51] and MoPtep1 [52]. In this study, a total of 372 effectors were predicted, in which many well-known effectors were found, such as MSP1 (G4MKI0) [15] and MPG1 (P52751) [53]. However, many secreted proteins without conserved Pfam domains or annotated as hypothetical proteins were also predicted as candidate effectors in our study, which can be the new effectors in *M. oryzae*. Previous studies have also reported this group of candidate effectors in some fungi considered to play a central role in establishing colonization and infection of plant tissue [54,55]. However, the majority of these effectors have not been experimentally tested for their functions in pathogenicity. Further investigation of the effector expression profiles during infection is needed to elucidate the roles of effectors during *M. oryzae* pathogenesis.

During the biotrophic phase, *M. oryzae* secretes a battery of effectors to suppress plant immunity and to facilitate fungal growth [56]. For example, AvrPiz-t suppresses rice immunity by affecting the flg22- and chitin-induced generation of reactive oxygen species and other defense responses [12]. Similarly, the effector Avr-Pii plays a role in the suppression of plant immunity via inhibition of Os-NADP-ME2. The effector MoPtep1 (peroxisomes-targeted effector protein 1) does not induce cell death but suppresses INF1-induced cell death in tobacco via the agrobacterium-mediated transient expression system in tobacco leaves [52]. Consistent with the results, our results showed that 16 of the selected 18 effectors suppressed BAX-induced cell death, but none of these effectors induced cell death when transiently expressed in *N. benthamiana*. However, previous studies also showed that some effectors induced cell death when transiently expressed in *N. benthamiana.* Nie et al. [57] identified that the effector MoVcpo from *M. oryzae* strongly induced ROS production and cell death in *N. benthamiana*. These results indicate that *M. oryzae* can employ multiple effectors to regulate the interaction with rice. Further investigations are needed to understand the action modes of different types of effectors and their synergistic role.

In summary, we performed a secretomic analysis of *M. oryzae* using an in vitro system, by spraying *M. oryzae* conidia onto the PVDF membrane to mimic the host–pathogen interaction at the early stages of infection. A high-quality *M. oryzae* secretome database including 3315 non-redundant secreted proteins was constructed using LC-MS/MS combined with a bioinformatics analysis; 84.5% of the secretome was identified as LSPs and 0.2% could not be predicted by these bioinformatics tools, which indicated the involvement of a complex secreted mechanisms in *M. oryzae*. Many new candidate effectors without conserved Pfam domains or annotated as hypothetical proteins were identified, although further direct experimental proof of protein function is needed. The *Agrobacterium*-mediated transient expression in *N. benthamiana* and qRT-PCR analysis showed that eighteen candidate effectors were truly functional proteins by experimental validation. Our study provides useful clues of the *M. oryzae* secretome, which enriches our understanding for further exploration of fungal pathogenicity. However, it is challenging to functionally characterize detailed information of the effectors about their functions in biological roles and in fungal virulence.

## 4. Materials and Methods

### 4.1. Fungi and Plants

*M. oryzae* race ZC13, one of the primary blast races in Guangdong Province, was cultured on CM medium (1% glucose, 0.5% peptone, 0.2% yeast extract, 0.1% casamino acids, 0.6% NaNO_3_, 0.05% KCl, 0.05% MgSO_4_, 1.5% KH_2_PO_4_) at 25 °C for 10 days. Fungal conidia were prepared following Li et al. [58]. The rice (*Oryza sativa* subsp. *indica*) cultivar CO39 was used in this study, which is susceptible to *M. oryzae*. Rice plants were maintained in a greenhouse at 25 ± 1 °C, 70–80% relative humidity with a 12 h photoperiod. Rice seedlings at fully fourth-leaf stage were used for all experiments. *N. benthamiana* were grown in the same conditions for 3 to 4 weeks and were used for all transient assays.

### 4.2. Extraction of Secreted Proteins

The secreted proteins were extracted according to the methods of Jung et al. [24] with some modifications. Briefly, the fungal conidial suspension (1 × 10^6^ conidia/mL) was sprayed onto PVDF membrane (Hybond-P, pore size 0.45 μM) and cultured at 28 °C in the dark. The PVDF membrane was washed twice with 5 mL of 10 mM Tris-HCl (pH 7.5) to collect the secreted proteins at 8 h and 24 h. To remove potential contamination from spores, germ tube, or appressorium, the collected samples were filtered through 0.45 μm filter membrane (Millipore, Tullagreen, Ireland) and then centrifuged at 18,500× *g* for 10 min. The supernatants were immediately mixed with 10 mM PMSF and 5 mM EDTA, which were used as crude protein solution. The supernatants were then concentrated by ultrafiltration using a 10 kDa membrane cutoff (Millipore, Tullagreen, Ireland). The residue solution was re-ultrafiltered with three volumes of Tris-HCl (pH 7.5), then transferred to a 3 kDa cutoff (Amicon Ultra-15 centrifugal filters, Millipore, Tullagreen, Ireland), and centrifuged for 20 min at 18,000× *g*. The supernatant was precipitated with acetone at −20 °C for 2 h. The precipitate was collected by centrifugation and dried by exposure to air. The precipitate was dissolved in a SDT lysis buffer (100 mM Tris-HCl, 1 mM DTT, 4% *w*/*v* SDS, pH 7.6). All the above procedures were carried out at 4 °C. Protein concentration was determined by the methods of Lowry et al. [59] with BSA as the standard. Three independent biological replicates were performed by pooling independent germinating conidia cultural samples.

### 4.3. Identification of Proteins by LC-MS/MS

The proteins from the 8 h and 24 h collections were mixed in a 1:1 (*w*/*w* total protein) ratio followed by digestion through the FASP procedure, as described previously [60]. LC-MS/MS analysis was performed on a Q Exactive mass spectrometer equipped with an EASY-Spray ion source (Thermo Fisher Scientific, Waltham, MA, USA), coupled to an Easy-nLC (Thermo Fisher Scientific) for 1 h. MS data acquired using a data-dependent top10 method dynamically choosing the most abundant precursor ions from the survey scan (300–1800 m/z) for HCD fragmentation were analyzed using MASCOT engine (Matrix Science, London, UK; version 2.4) against the UniProtKB *M. oryzae* database. For protein identification, the following options were used: trypsin cleavage, double missed cleavage, peptide mass tolerance set to 20 ppm, MS/MS tolerance set to 0.1 Da, carbamido methylation set as fixed modification, FDR ≤ 0.01.

### 4.4. Bioinformatics Analysis of the M. oryzae Secreted Proteins

Classically secreted proteins were predicted as described previously [43]. The signal peptides and signal peptide cleavage sites were predicted by SignalP 5.0 (https://services.healthtech.dtu.dk/service.php?SignalP-5.0 accessed on 10 June 2022) [61]. Subcellular localization predictions were analyzed using the Fungi model of WoLF PSORT (https://wolfpsort.hgc.jp/ accessed on 14 June 2022) and Target P (https://services.healthtech.dtu.dk/service.php?TargetP-2.0 accessed on 16 June 2022) [62]. The transmembrane domains were predicted using TMHMM 2.0 (https://services.healthtech.dtu.dk/service.php?TMHMM-2.0 accessed on 18 June 2022) [63]. The glycosylphosphatidylinositol (GPI) anchor site was predicted by the Big-PI Fungal Predictor server (https://mendel.imp.ac.at/gpi/fungi_server.html accessed on 19 June 2022) [64]. The SecretomeP 2.0 server (https://services.healthtech.dtu.dk/service.php?SecretomeP-2.0 accessed on 23 October 2022) was used to predict non-classical secreted proteins lacking signal peptides (NN-score > 0.6) [65]. 

### 4.5. Functional Annotation of Secreted Proteins 

EggNOG-mapper v5.0 (http://eggnog5.embl.de/#/app/home accessed on 16 November 2022), Blast2GO (https://www.blast2go.com/ accessed on 18 November 2022), and KEGG Automatic Annotation Server (http://www.genome.jp/kegg/kaas/ accessed on 18 November 2022) were used to generate COG, GO, and KEGG orthology (KO) annotations for the secreted proteins, respectively [66,67,68].

### 4.6. Prediction of CAZymes and Effectors

CAZymes were predicted by the dbCAN2 meta server (http://bcb.unl.edu/dbCAN2/ accessed on 23 November 2022) using all three available tools, including HMMER (e-value < 1 × 10^−15^, coverage > 0.35), DIAMOND (e-value < 1 × 10^−102^), and Hotpep (frequency > 2.6, hits > 6). To identify the candidate effectors, two approaches were used: (1) EffectorP 3.0 (https://effectorp.csiro.au/ accessed on 30 November 2022) was used for effector prediction; (2) secreted small cysteine-rich proteins (≤300 amino acids and ≥4 cysteine residues) were considered candidate effectors [69].

### 4.7. Quantitative Reverse Transcription (qRT-PCR) Analysis

Total RNA was extracted using a Fungal RNA kit or a Plant RNA kit (Omega Bio-tek, Norcross, GA, USA) according to the manufacturer’s instructions. RNA was reversely transcripted using the PrimeScript TM RT Master Mix Kit (TaKaRa, Beijing, China) following the manufacturer’s instructions. Specific primers for qRT-PCR analysis were designed using Primer Premier 5.0 software (Table S4). The qRT-PCR was conducted on a CFX Coxnnect^TM^ Real-Time System (Bio-Rad, Hercules, CA, USA) with the SYBR Premix Ex Taq Kit (TaKaRa, Beijing, China) according to the manufacturer’s instructions. The actin gene was used as a reference. Each sample was represented by three biological replicates. Relative transcript levels for each gene were calculated using the formula 2^−ΔΔct^ [70].

### 4.8. Transient Expression of Candidate Effectors in N. benthamiana

The open reading frames of eighteen candidate effectors, fused to HA-tag at N-terminus, were cloned into the PBI121 vector. The recombinant vectors were transformed into *Agrobacterium tumefaciens* strain GV3101 through the electroporation method and then transiently expressed in 4-week-old *N. benthamiana* leaves, as described previously [71]. For the induction assay of cell death, the *A. tumefaciens* cell suspensions carrying an empty vector (pBI121), a vector carrying BAX (Bcl2-associated X protein), or vectors carrying the candidate effectors were infiltrated separately into *N. benthamiana* leaves. For the suppression assay of cell death, the *A. tumefaciens* suspensions carrying pBI121, a vector carrying TCTP (translationally controlled tumor protein), or vectors carrying the candidate effectors were separately infiltrated; 24 h later, *A. tumefaciens* suspensions containing a BAX-carrying vector were infiltrated at the same location. pBl121 and the vector carrying TCTP were used as negative controls, while the vector carrying BAX was used as a positive control. Each assay was performed on five leaves from three individual plants and repeated at least three times. Leaf phenotypes were photographed 3–4 d after infiltration. 

### 4.9. Statistical Analysis

Statistical analyses were performed using the ANOVA of the software SPSS 13.0 for Windows (SPSS Inc., Chicago, IL, USA). Differences among means were analyzed using Duncan’s multiple range tests. To determine the significant difference among group means, data are presented as means ± standard error (SE).

## Data Availability

Not applicable.

## Supplementary Materials

The following supporting information can be downloaded at https://www.mdpi.com/article/10.3390/ijms24043189/s1.
