# Supplementary material for: Screening of Candidate Effectors from Magnaporthe oryzae by In Vitro Secretomic Analysis
Source: Int J Mol Sci. 2023 Feb 6;24(4):3189. doi: 10.3390/ijms24043189 (PMC9962664; doi:10.3390/ijms24043189)
Supplement: Supplementary file 1 [file ijms-24-03189-s001.zip › Li et al_Supplementary Table S4.pdf]

Table S4. Primers used in this study.

Uniprot No.	Gene name	Primer name	Primer Sequence (5'-3')
**Construction for transient expression vector**			
G4NG69	MGG_14966	MGG_14966-F	GCTCTAGAATGTACCCATACGATGTTCCAGATTACGCTGGCATGAAGTACACCACTTTCGCTGC
		MGG_14966-R	CGAGCTCTTAACTTAAGAGCAGGTGCCGAA
		NSP-MGG-14966-F	GCTCTAGAATGTACCCATACGATGTTCCAGATTACGCTATGATTCCCTCTGCTCTGGCC
		NSP-MGG-14966-R	CGAGCTCTTAAGAGCAGGTGCCGAATGC
G4NIH0	MGG_09842	MGG_09842-F	GAGAACACGGGGGACTCTAGAATGTACCCATACGATGTTCCAGATTACGCTGGC ATGCAGTTTCTCAGCACCATGC
		MGG_09842-R	CGATCGGGGAAATTCGAGCTCTTACCAGTCCCAGCACTGGTAGC
		NSP-MGG-09842-F	GAGAACACGGGGGACTCTAGAATGTACCCATACGATGTTCCAGATTACGCTGGC ATCCCGGCCGAGCTC
		NSP-MGG-09842-R	CGATCGGGGAAATTCGAGCTCTTACCAGTCCCAGCACTGGTAGC
G4MVY9	MGG_15924	MGG_15924-F	GCTCTAGAATGTACCCATACGATGTTCCAGATTACGCTGGCATGCGCACATTTGCTATTCTC
		MGG_15924-R	CGAGCTCTTAGACGCATCCAATAAATCCCTT
		NSP-MGG-15924-F	GCTCTAGAATGTACCCATACGATGTTCCAGATTACGCTATGTGGCCTGGTTGCCGC
		NSP-MGG-15924-R	CGAGCTCTTAGACGCATCCAATAAATCCCT
G4NAI7	MGG_13654	MGG_13654-F	GAGAACACGGGGGACTCTAGAATGTACCCATACGATGTTCCAGATTACGCTGGCATGAAGTTCCTCGGCCTCACC
		MGG_13654-R	CGATCGGGGAAATTCGAGCTCCTAGGTGCAGTTGGTGCAAGTG
		NSP-MGG-13654-F	GAGAACACGGGGGACTCTAGAATGTACCCATACGATGTTCCAGATTACGCTATGTCCCGCTGGCCATGG
		NSP-MGG-13654-R	CGATCGGGGAAATTCGAGCTCCTAGGTGCAGTTGGTGCAAGTG
Q2KEU7	MGG_09134	MGG_09134-F	GCTCTAGAATGTACCCATACGATGTTCCAGATTACGCTATGCAGATCAAGGCCCTCA
		MGG_09134-R	CGAGCTCTTAATGGCCGATGGGTGC
		NSP-MGG_09134-F	GCTCTAGAATGTACCCATACGATGTTCCAGATTACGCTGGCATGCCCACCGACCCG
		NSP-MGG_09134-R	CGAGCTCTTAATGGCCGATGGGTGC
G4NFX3	MGG_11072	MGG_11072-F	GCTCTAGAATGTACCCATACGATGTTCCAGATTACGCTATGCAGATCAAGACTTTCGC

		MGG_11072-R	CGAGCTC TTAGTAGGTGCAAGTGCACTCG
		NSP-MGG_11072-F	GCTCTAGAATGTACCCATACGATGTTCCAGATTACGCTGGCCTCTTCATCGCCTTCGCTG
		NSP-MGG_11072-R	CGAGCTCTTAGTAGGTGCAAGTGCACTCG
G4MWC0	MGG_08373	MGG_08373-F	GCTCTAGAATGTACCCATACGATGTTCCAGATTACGCTATGCAGTTCTCAGCACTTCTCG
		MGG_08373-R	CGAGCTCCTAAGCACTAATTGAGCGGCG
		NSP-MGG_08373-F	GCTCTAGAATGTACCCATACGATGTTCCAGATTACGCTGGCGTGTCGGCCATCGATGTCA
		NSP-MGG_08373-R	CGAGCTCCTAAGCACTAATTGAGCGGCG
G4NJJ0	MGG_02638	MGG_02638-F	GCTCTAGAATGTACCCATACGATGTTCCAGATTACGCTATGCTGCTCAACCACACCAA
		MGG_02638-R	CGAGCTCTCATCCACACAGCCCCGT
		NSP-MGG_02638-F	GCTCTAGAATGTACCCATACGATGTTCCAGATTACGCTGGCGGCAAAATCGAGGGCAATC
		NSP-MGG_02638-R	CGAGCTCTCATCCACACAGCCCCGT
G4N4K5	MGG_05982	MGG_05982-F	GCTCTAGAATGTACCCATACGATGTTCCAGATTACGCTGGCATGAAGCTTTTCACTATAATTATGG
		MGG_05982-R	CGAGCTCTTACCGGACACGGTAGCACTT
		NSP-MGG_05982-F	GCTCTAGAATGTACCCATACGATGTTCCAGATTACGCTGGCGCACCGGTCGAGAGCACAA
		NSP-MGG_05982-R	CGAGCTCTTACCGGACACGGTAGCACTT
G4MPP1	MGG_02234	MGG_02234-F	GCTCTAGAATGTACCCATACGATGTTCCAGATTACGCTGGCATGCACTTCCCCACCATCCTC
		MGG_02234-R	CGAGCTCCTATTCCTCGGAGCTGCG
		NSP-MGG_02234-F	GCTCTAGAATGTACCCATACGATGTTCCAGATTACGC GGCTCAACGTCCGTTCGCAC
		NSP-MGG_02234-R	CGAGCTCCTATTCCTCGGAGCTGCG
G4N4A2	MGG_05943	MGG_05943-F	GAGAACACGGGGGACTCTAGAATGTACCCATACGATGTTCCAGATTACGCTGGCATGCAGCTCACCAAGGTCTT
		MGG_05943-R	CGATCGGGGAAATTCGAGCTCTCAGCTGCAGTTGAAAGGG
		NSP-MGG_05943-F	CGATCGGGGAAATTCGAGCTCATGTACCCATACGATGTTCCAGATTACGCTGGCGCCTTTGTGATCCCCGC
		NSP-MGG_05943-R	CGATCGGGGAAATTCGAGCTCTCAGCTGCAGTTGAAAGGG
G4NDA8	MGG_00269	MGG_00269-F	GCTCTAGAATGTACCCATACGATGTTCCAGATTACGCTGGCATGCTTTCACTCTCCATCGTCAC
		MGG_00269-R	CGAGCTCCTAGCTGGGGATACACATGAAGGA
		NSP-MGG_00269-F	GCTCTAGAATGTACCCATACGATGTTCCAGATTACGCTGGCGCCCCGGCGGAGGTCA

		NSP-MGG_00269-R	CGAGCTCCTAGCTGGGGATACACATGAAGGA
Q2KHE0	MGCH7_ch7g45	MGCH7_ch7g45-F	GCTCTAGAATGTACCCATACGATGTTCCAGATTACGCTGGCATGCATTTCCCCTCCGTCTTAGC
		MGCH7_ch7g45-R	CGAGCTCCTAGGGGTTGGTAGCGTTTGGG
		NSP-MGCH7_ch7g45-F	GCTCTAGAATGTACCCATACGATGTTCCAGATTACGCTGGCGCGCCAATGCTATCCGAG
		NSP-MGCH7_ch7g45-R	CGAGCTCCTAGGGGTTGGTAGCGTTTGG
G4N7U6	MGG_06359	MGG_06359 -F	GAGAACACGGGGGACTCTAGAATGTACCCATACGATGTTCCAGATTACGCTGGCATGCGCGCACCCACAG
		MGG_06359 -R	CGATCGGGGAAATTCGAGCTCTCAAGTCTTGATGGTCAGCTTCATA
		NSP-MGG_06359 -F	GAGAACACGGGGGACTCTAGAATGTACCCATACGATGTTCCAGATTACGCTGGCGCACCCTCGAGGAACATATACG
		NSP-MGG_06359 -R	CGATCGGGGAAATTCGAGCTCTCAAGTCTTGATGGTCAGCTTCATA
G4N8U1	MGG_10105	MGG_10105-F	GCTCTAGAATGTACCCATACGATGTTCCAGATTACGCTGGCATGCAGATCAAGACCCTCATCG
		MGG_10105-R	CGAGCTCTTAGGCTCCGACTGGGGC
		NSP-MGG_10105-F	GCTCTAGAATGTACCCATACGATGTTCCAGATTACGCTGGCATGCCCACCGACCCGC
		NSP-MGG_10105-R	CGAGCTCTTAGGCTCCGACTGGGGC
G4MMD6	MGG_01993	MGG_01993-F	GCTCTAGAATGTACCCATACGATGTTCCAGATTACGCTGGCATGAAGTTCACCCTTCCCATCA
		MGG_01993-R	CGAGCTCTCAAGTGGCACAGCACTTGTAG
		NSP-MGG_01993-F	GCTCTAGAATGTACCCATACGATGTTCCAGATTACGCTGGCGCCATTGGGGACCTCTGC
		NSP-MGG_01993-R	CGAGCTCTCAAGTGGCACAGCACTTGTAG
G4MKE6	MGG_10531	MGG_10531-F	GCTCTAGAATG TACCCATACGATGTTCCAGATTACGCTGGCATGCAGCTCATCATCTTCCTCA
		MGG_10531-R	CGAGCTCTTACTCCTGGTCGAGAAGTGC
		NSP-MGG_10531-F	GCTCTAGAATGTACCCATACGATGTTCCAGATTACGCTGGCGCCGACGTCTGCGGC
		NSP-MGG_10531-R	CGAGCTCTTACTCCTGGTCGAGAAGTGC
G5EH55	MGCH7_ch7g5	MGCH7_ch7g5-F	GCTCTAGAATGTACCCATACGATGTTCCAGATTACGCTGGCATGCTCCCAGGTAACCTTTTTA
		MGCH7_ch7g5-R	CGAGCTCTTAACGTGGTCCGTAATGGTAG
		NSP-MGCH7_ch7g5-F	GCTCTAGAATGTACCCATACGATGTTCCAGATTACGCTGGCATGCCCCCTTCTCACAAAC
		NSP-MGCH7_ch7g5-R	CGAGCTCTTAACGTGGTCCGTAATGGTAG

qRT-PCR analysis of *M. oryzae* candidate effector genes

G4NGZ4	MGG_03982	qMoActin-F	TCGACGTCCGAAAGGATCTGT
(MoActin)		qMoActin-R	ACTCCTGCTTCGAGATCCACATC
G4NIH02	MGG_09842	qMGG_09842-F	CGTCTACCTGTGCACCAACT
		qMGG_09842-R	TGGTCAGGTCAAAGTGCCAG
G4MVY9	MGG_15924	qMGG_15924-F	GCATCCGACCCACTTGATGA
		qMGG_15924-R	ACCTGTTGAACTACGCGTCC
G4NAI7	MGG_13654	qMGG_13654-F	TACGTCCGGAACATTGGCTC
		qMGG_13654-R	GCAGTTGGTGCAAGTGACA
Q2KEU75	MGG_09134	qMGG_09134-F	ACTGACATCCTCGGTCTTGC
		qMGG_09134-R	GCAGAGAAGTGCCTGTCCAA
G4NFX3	MGG_11072	qMGG_11072-F	GAGACCTCCACCTTCAGGGA
		qMGG_11072-R	TGATGTCGTTGGACTTGGGG
G4MWC0	MGG_08373	qMGG_08373-F	ACCTCAACCCCAACGTATGC
		qMGG_08373-R	AAGTCTCTGCCGTTGGAGTC
G4NJJ0	MGG_02638	qMGG_02638-F	CCGACTTGCTGCACAACTTT
		qMGG_02638-R	CCACTTGCTCTCAACGCAAC
G4MPP1	MGG_02234	qMGG_02234-F	ACATCGCCATCGCCTACAC
		qMGG_02234-R	ACGAGTTGTACCAGCGTCC
G4N4A2	MGG_05943	qMGG_05943-F	AAGTATGGCCAGCCTCTGGG
		qMGG_05943-R	AGTTGAAAGGGACCCGAGTG
G4NDA8	MGG_00269	qMGG_00269-F	GACTGGGCTTCTTTCTACGG
		qMGG_00269-R	CGTTGCAGTTGTAGCGAACG
Q2KHE0	MGCH7_ch7g4 5	qMGCH7_ch7g45-F	GACTAGCGCTAAAAACGGCG
		qMGCH7_ch7g45-R	CTGGTCCAGTTGATCCCGTC
G4N7U6	MGG_06359	qMGG_06359-F	AGGCAAATGATCAGCCCACA

		qMGG_06359-R	CAAGGGCTCTCGTGGAAGTT
G4MMD6	MGG_01993	qMGG_01993-F	CCTTCTGTAGCAACCGTGGA
		qMGG_01993-R	CCCCAGTGGACATACAAGACC
G4MKE6	MGG_10531	qMGG_10531-F	CTCGCTGGTCAAGGTCCG
		qMGG_10531-R	CTCCTGGTCGAGAAGTGCAG
G5EH55	MGCH7_ch7g5	qMGCH7_ch7g5-F	GCCCCCTTCTCACAAACCTT
		qMGCH7_ch7g5-R	TAGAAGGTGGGTCGTCGCTA
G4NG69	MGG_14966	qMGG_14966-F	CATCACCCAGCAGTTGACCA
		qMGG_14966-R	TGGCAGTACGGATCATGTGG
G4N8U1	MGG_10105	qMGG_10105-F	CATCGTCGCCCTCTTTGCC
		qMGG_10105-R	CCTCGCACGCGTCATAGT
G4N4K5	MGG_05982	qMGG_05982-F	AAGAAGCGCTACGAGGTTCAA
		qMGG_05982-R	TCCTCACCGGGCCTTATGT
