# Supplementary material for: Screening of Candidate Effectors from Magnaporthe oryzae by In Vitro Secretomic Analysis
Source: Int J Mol Sci. 2023 Feb 6;24(4):3189. doi: 10.3390/ijms24043189 (PMC9962664; doi:10.3390/ijms24043189)
Supplement: Supplementary file 1 [file ijms-24-03189-s001.zip › R1_Li et al_Figure S1-S7_20230201.pdf]

Supplementary Figures

Screening of candidate effectors from *Magnaporthe oryzae* by *in vitro* secretomic analysis

Figure S1.

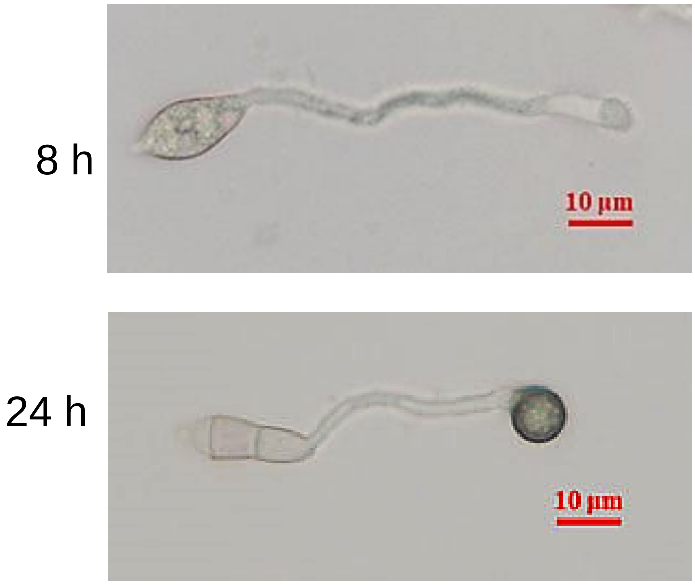

Figure S1. Microscopic observation of *M. oryzae* spore germination at 8, and 24 h. *M. oryzae* spores were sprayed on a hydrophobic PVDF membrane by an air compressor. Microscopic observation was performed at 300× magnification.

Figure S2.

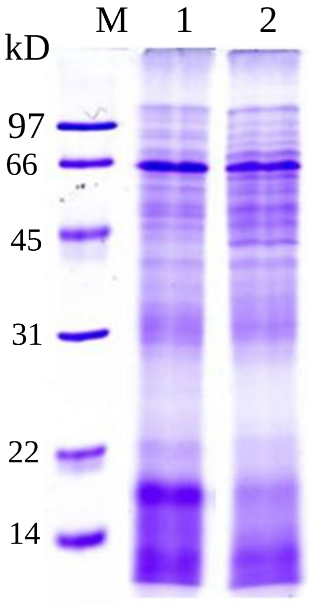

Figure S2. SDS-PAGE analysis of the secreted proteins of *M. oryzae*. The gel was stained with CBB to visualize total proteins. Ten μg total proteins per lane were loaded. Lane M, protein marker; lane 1 and 2, the secreted proteins isolated at 8 h and 24 h, respectively.

Figure S3.

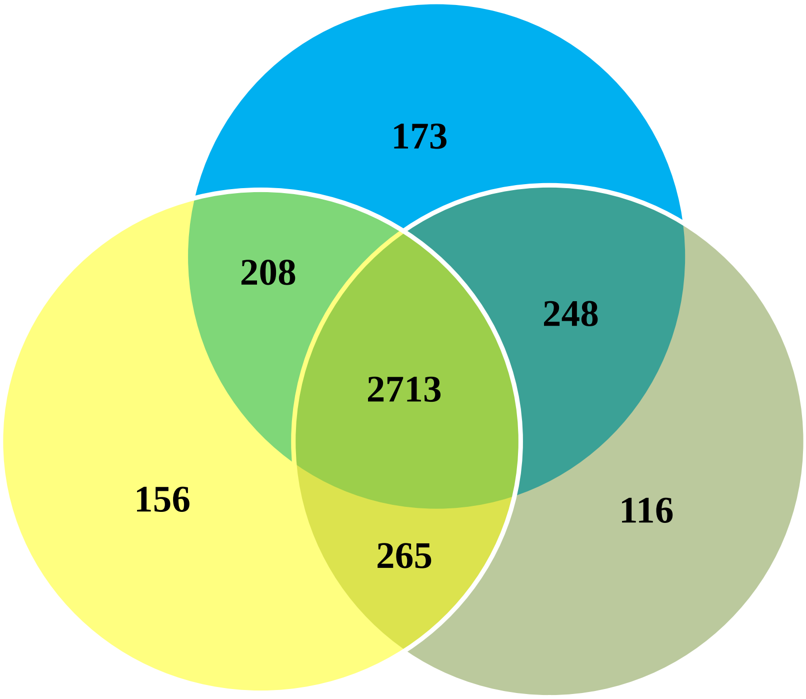

Figure S3. Venn diagram analysis of the secreted proteins of *M. oryzae* that overlapped between three biological replicates.

Figure S4.

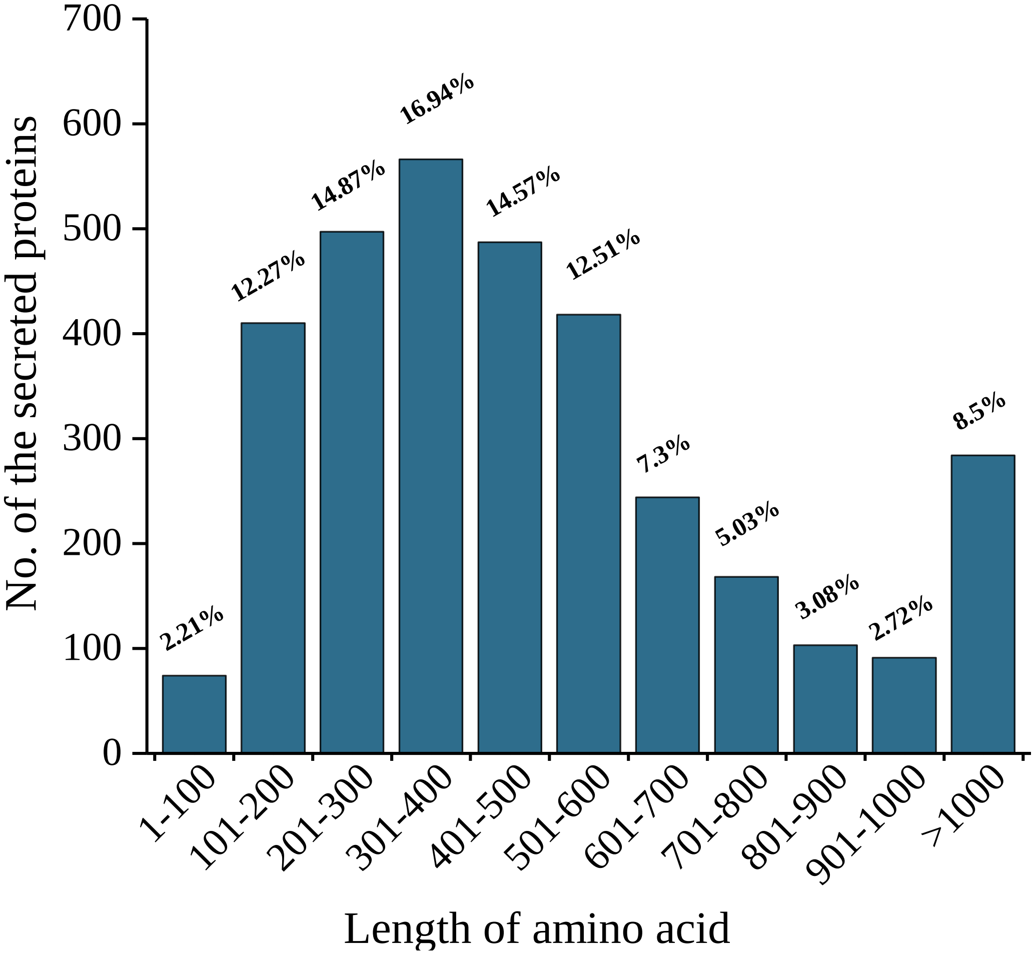

Figure S4. The amino acid length distribution of the secreted proteins in *M. oryzae*.

Figure S5.

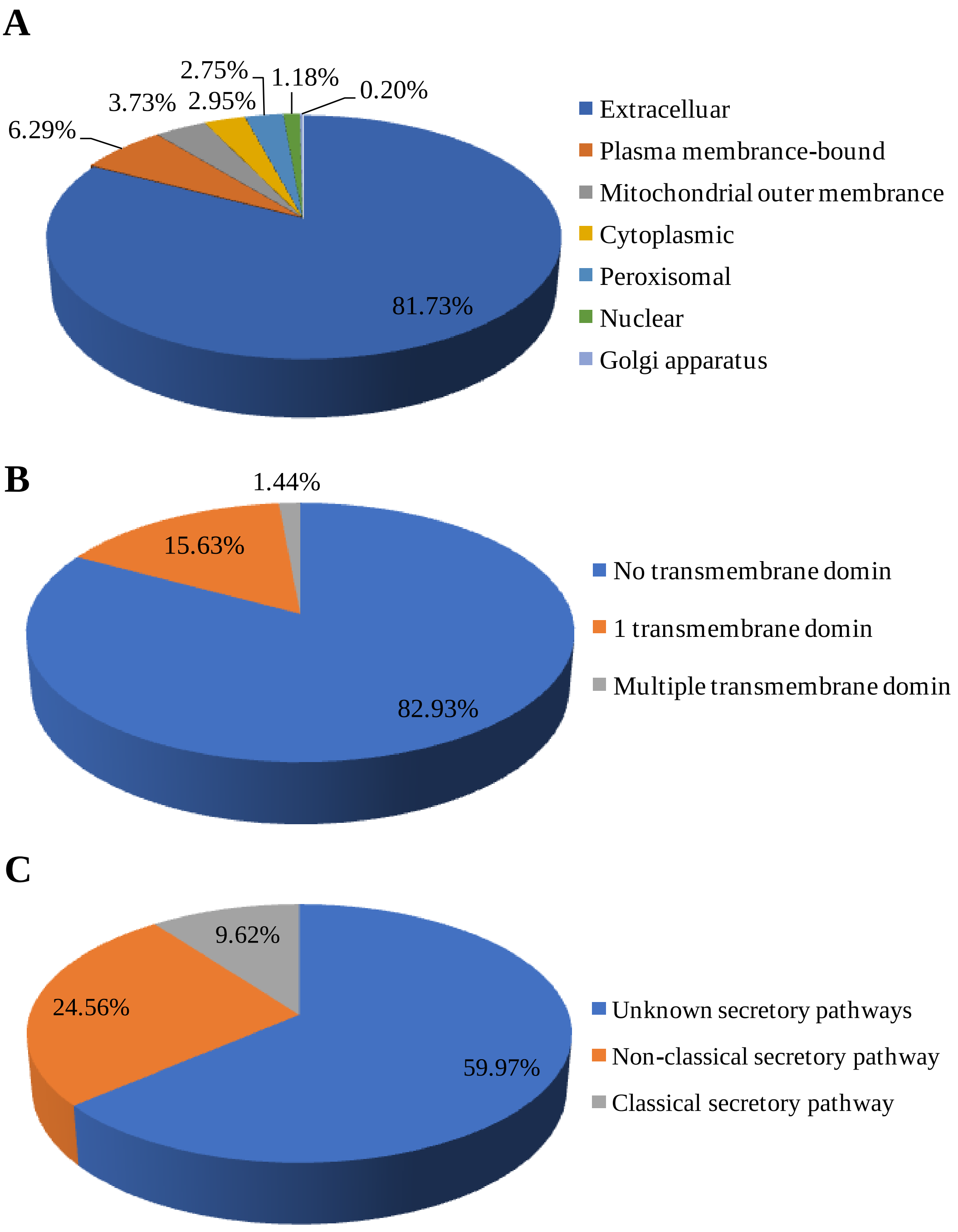

Figure S5. The secretion pathways analysis of *M. oryzae* secretome. (a) Subcellular location prediction of the secreted proteins by WOLF PSORT and TargetP. (b) The transmembrane prediction of the extracellular proteins by TMHMM. (c) Secretory pathways of *M. oryzae* secreted proteins.

Figure S6.

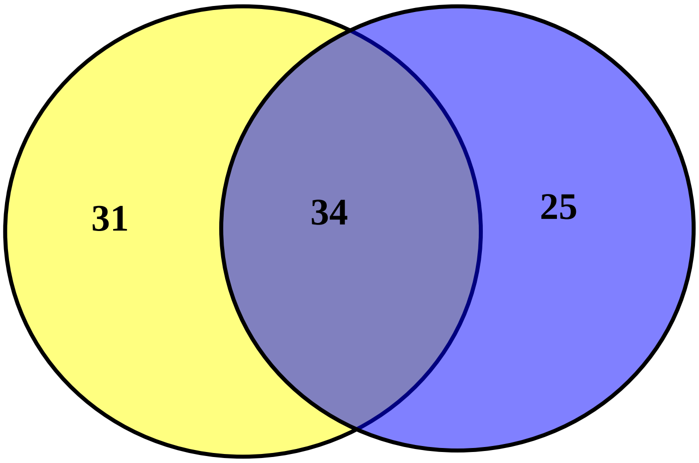

Figure S6. Venn diagram analysis of the candidate effectors of *M. oryzae* that overlapped between two predication approaches. Yellow, candidate effectors predicted by EffectorP 3.0; Blue, candidate effectors predicted by the criteria of amino acid length ≤300 and cysteine number ≥ 4.

Figure S7.

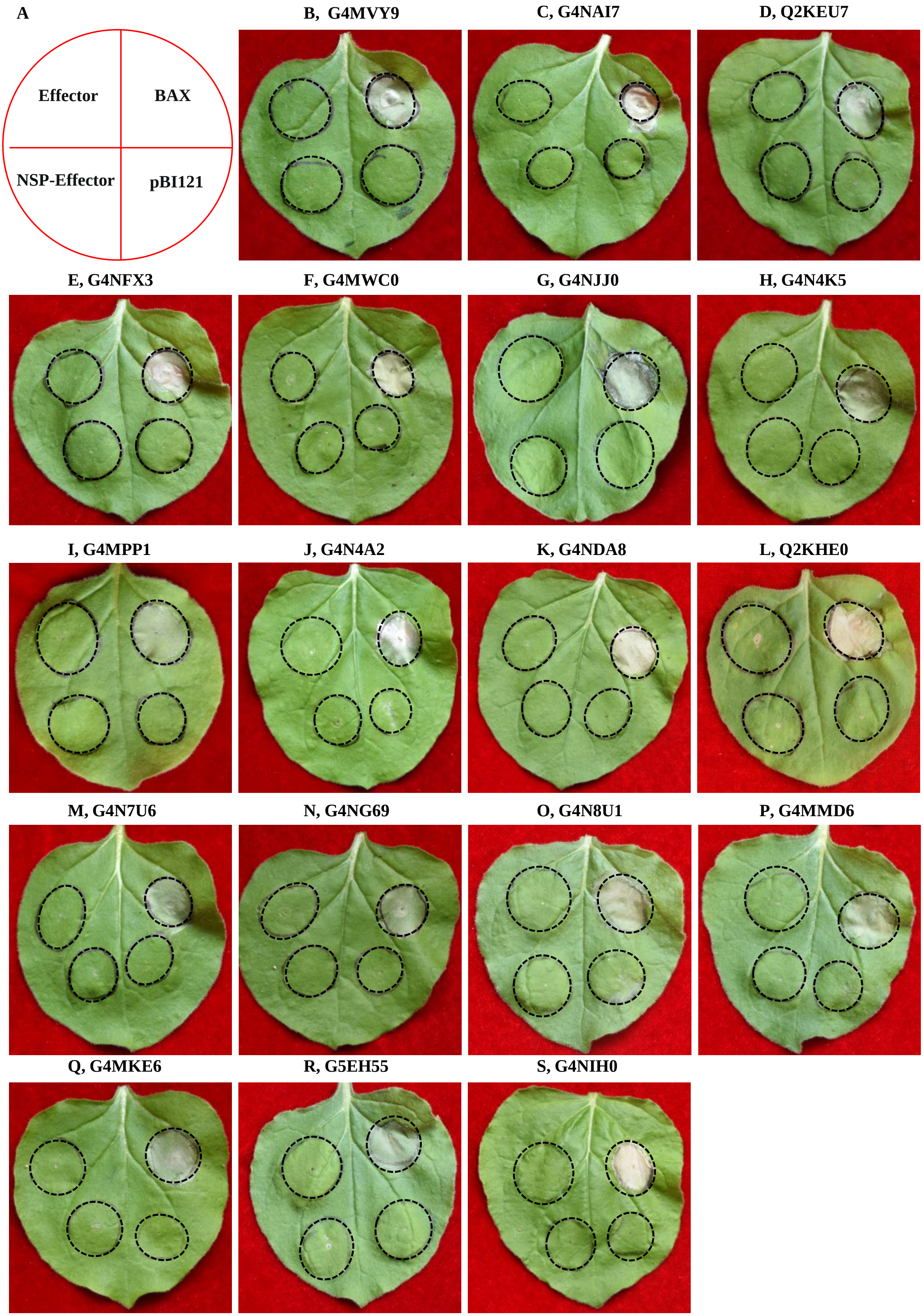
A
Effector
BAX
NSP-Effector
pBI121
B, G4MVY9
C, G4NAI7
D, Q2KEU7
E, G4NFX3
F, G4MWC0
G, G4NJJ0
H, G4N4K5
I, G4MPP1
J, G4N4A2
K, G4NDA8
L, Q2KHE0
M, G4N7U6
N, G4NG69
O, G4N8U1
P, G4MMD6
Q, G4MKE6
R, G5EH55
S, G4NIH0

Figure S7. Transient expression of *M. oryzae* candidate effectors can not induce cell death in *N. benthamiana* leaves using agroinfiltration. *N. benthamiana* leaves were infiltrated with *A. tumefaciens* carrying pBI121 empty vector, the BAX gene, or candidate effector genes. BAX was used as positive control, and pBI121 empty vector was used as negative control. The leaves were photographed at 5 d post-infiltration. (A) Schematic diagram showing injection on *N. benthamiana* leaves. (B):G4MVY9; (C):G4NAI7; (D):Q2KEU7; (E):G4NFX3; (F):G4MWC0; (G):G4NJJ0; (H):G4N4K5; (I):G4MPP1; (J):G4N4A2; K):G4NDA8; (L):Q2KHE0: (M):G4N7U6; (N):G4NG69; (O):G4N8U1; (P):G4MMD6; (Q):G4MKE6; (R):G5EH55 and (S):G4NIH0.
